# Debatte über die Doppik: Replik und Erwiderung

**DOI:** 10.1007/s10273-021-2856-y

**Published:** 2021-02-20

**Authors:** Stephan Stüber, Désirée I. Christofzik, Florian Dorn, Stefanie Gäbler, Christian Raffer, Felix Rösel

**Affiliations:** 1 Referat Haushalts- und Kassenrecht, Haushaltssystematik, Finanzbehörde der Freien und Hansestadt Hamburg, Gänsemarkt 36, 20354 Hamburg, Deutschland; 2 Zentralbereich / öffentliche Finanzwirtschaft, Hochschule des Bundes für öffentliche Verwaltung in Brühl, Willy-Brandt-Straße 1, 50321 Brühl, Deutschland; 3 öffentliche Finanzen und politische ökonomie, ifo Institut München, Poschingerstr. 5, 81679 München, Deutschland; 4 Heroldstr. 18, 01157 Dresden, Deutschland; 5 Hertie School Berlin, Friedrichstraße 180, 10117 Berlin, Deutschland; 6 ifo Institut Dresden, Einsteinstrasse 3, 01069 Dresden, Deutschland

**Keywords:** H83, H71, H72

## Abstract

In der Septemberausgabe 2020 veröffentlichte der Wirtschaftsdienst einen Aufsatz mit dem Titel „Bremst die Doppik öffentliche Investitionen? Ergebnisse aus drei aktuellen Evaluationsstudien“ von Désirée Christofzik, Florian Dorn, Stefanie Gäbler, Christian Raffer und Felix Rösel. Stephan Stüber vertritt in einer Replik eine andere Auffassung, im Anschluss erläutern Christofzik et al. ihren Standpunkt in einer Erwiderung.

## Abstract (en)

In its September 2020 issue, Wirtschaftsdienst published an article entitled “Bremst die Doppik öffentliche Investitionen? Ergebnisse aus drei aktuellen Evaluationsstudien” by Désirée Christofzik, Florian Dorn, Stefanie Gäbler, Christian Raffer and Felix Rösel. Stephan Stüber takes a different view in a reply, and Christofzik et al. explain their point of view in a response.

### Replik: Die Doppik bremst keine öffentlichen Investitionen!

In ihrem Beitrag in der Septemberausgabe 2020 zeigen sich Désirée Christofzik, Florian Dorn, Stefanie Gäbler, Christian Raffer und Felix Rösel angesichts mehrerer Studien zur Umstellung des Rechnungswesens in Kommunen von der Kameralistik auf die Doppik davon überrascht, dass „tendenziell die Investitionsausgaben wie etwa für Baumaßnahmen oder den Erwerb von Grundstücken und Gebäuden“ sinken. Und weiter heißt es: „Wenngleich diese Effekte nicht in allen Studien oder Spezifikationen statistisch signifikant sind, so ist die übereinstimmende Beobachtung eines negativen Effekts auf die öffentlichen Investitionen doch bemerkenswert – lag doch eine Hoffnung auf einer Stärkung der Vermögenssubstanz.“ Die Ursachenanalyse liest sich wie folgt: „Eine Ursache könnten die höheren Verwaltungsausgaben und die geringeren Einnahmen aus der Veräußerung von öffentlichem Vermögen sein, die den finanziellen Spielraum für neue Investitionen reduzieren. Kommunale Beschäftigte und politische Entscheidungsträger könnten außerdem vor Investitionen zurückschrecken, da sie um den Zusatzaufwand bei der Vermögensbewertung und die Abschreibungen im doppischen System wissen, die sich negativ auf folgende Haushalte auswirken. Auch die fehlende Erfahrung mit dem neuen Buchungssystem könnte eine Zurückhaltung bei der Investitionsneigung erklären. Schließlich könnte die erhoffte höhere Transparenz des Haushalts dazu geführt haben, dass ein Bedarf eben nicht bei neuen Investitionen, sondern der Unterhaltung des bestehenden Vermögens aufgedeckt wurde.“ Empfohlen wird sodann, die Bewertung der Nachhaltigkeit von Haushaltsentscheidungen nicht allein an der Entwicklung der Investitionen festzumachen und die Hintergründe des Investitionsrückgangs unter der Doppik noch näher zu beleuchten. Die Auswertung der Studien führt die Autor:innen zu dem Schluss, es sei unklar, ob eine Umstellung von Länder- und Bundeshaushalten auf doppische Standards die öffentlichen Investitionen in Deutschland nicht sogar zusätzlich ausbremsen könnte. Die in der Schlussfolgerung enthaltene These – wenn nach Auffassung der Autor:innen auch noch näher zu prüfen – ist so steil, dass sie nicht unwidersprochen bleiben kann. Wer in seiner Studie auf ein „überraschendes Ergebnis“ stößt, sollte sich gehalten fühlen, nach plausiblen Erklärungen zu suchen statt zu spekulieren.

Die Freie Hansestadt Hamburg hat mit dem Haushaltsjahr 2015 ihr Haushalts- und Rechnungswesen auf einen doppischen Produkthaushalt umgestellt. Das neue Haushaltswesen hat Politik und Verwaltung einiges abverlangt. Ohne dies im Einzelnen ausführen zu können, kann sicher auch festgehalten werden, dass die Doppik das Entscheidungsverhalten verändert hat. Dass aber Verwaltung

**Dr. Stephan Stüber** leitet das Referat Haushalts- und Kassenrecht, Haushaltssystematik der Finanzbehörde der Freien und Hansestadt Hamburg.

und Politik vor „Investitionen zurückschrecken, da sie um den Zusatzaufwand bei der Vermögensbewertung und die Abschreibungen im doppischen System wissen, die sich negativ auf folgende Haushalte auswirken“ und dann auch noch stattdessen Aufwendungen planen und verursachen, dürfte niemand beobachtet haben können. Im Gegenteil: Ich nehme Politiker wahr, die die Umstände in der Kommune, in der sie leben, gestalten wollen. Von „technischen“ Fragen wie einer Vermögensbewertung lassen sie sich dabei nicht abschrecken.

Für die in den dargestellten Studien getroffenen Feststellungen liegen zahlreiche plausiblere Gründe auf der Hand, als derjenige, den die Autor:innen heranziehen:

1. Es geht schon mit den gewählten Begriffen los, wenn im Zusammenhang mit der Doppik von „Investitionsausgaben“ oder davon gesprochen wird, mit der Umstellung auf eine doppische kommunale Haushaltsführung seien in der Tendenz höhere „laufende Verwaltungsausgaben verbunden, z.B. für Personal oder Sachaufwand.“ So sind (kamerale) Verwaltungsausgaben und (doppischer) Verwaltungsaufwand (insbesondere Personal- und Sachaufwand) weder begrifflich noch der Sache nach dasselbe. In der Doppik müssen die laufenden Sachaufwendungen im Vergleich zu den kameralen Sachausgaben steigen. Denn schließlich umfassen diese im Unterschied zur Kameralistik auch nicht zahlungswirksame Vorgänge wie insbesondere Abschreibungen auf das Vermögen. Der Personalaufwand enthält unter anderem Zuführungen zu den Rückstellungen für Pensionen. Unklar bleibt in der Darstellung, ob die „doppischen Verwaltungsausgaben“ um solche Effekte bereinigt wurden. Sollte es diese Bereinigung nicht gegeben haben, muss es sehr verwundern, dass der Personalaufwand im Vergleich zu den (kameralen) Personalausgaben nicht signifikant gestiegen ist.

Auch erwecken die gewählten Begriffe zumindest den Eindruck, als würden (kamerale) Investitionsausgaben mit (doppischen) Investitionsauszahlungen gleichgesetzt. Die Investitionsbegriffe der Doppik und der Kameralistik unterscheiden sich aber, wenn auch nach den haushaltssystematischen Vorschriften für die Kommunen z.B. in Bayern[1] möglicherweise nicht so stark wie auf der staatlichen Ebene.[2] Aber zumindest folgende Unterschiede dürften auch für die Kommunen festzustellen sein:

a) Nach den kameralen Vorschriften stellt der Erwerb einer Beteiligung oder einer Kapitaleinlage eine Investition dar. Das entspricht den Regelungen für die Doppik. Nur musste in der Haushaltspraxis der Freien und Hansestadt Hamburg gelegentlich Folgendes festgestellt werden: Im Haushaltsplan wurde eine Kapitaleinlage als Investitionsauszahlung veranschlagt und dann später auch so gebucht. Die kamerale Betrachtung endet nun – die Kapitaleinlage bleibt eine Kapitaleinlage und damit eine Investition. In der Doppik wird am Ende des Haushaltsjahres für den Jahresabschluss nachgesehen, ob das Eigenkapital der Beteiligung in Höhe der Kapitaleinlage gestiegen ist. Ist das nicht der Fall, handelt es sich nicht (mehr) um eine Kapitaleinlage, sondern im Ergebnis um einen Betriebsmittelzuschuss. Die vermeintliche Investition entpuppt sich als konsumtive Maßnahme (siehe z.B. Mitteilung des Senats an die Bürgerschaft, 2018).

b) Die Umstellung auf die Doppik hat in Hamburg viele Sachverhalte zutage gefördert, die während der Kameralistik den Investitionen zugeordnet wurden, obwohl dies bei genauerer Betrachtung schon damals kaum vertretbar war (siehe z.B. Rechnungshof der Freien und Hansestadt Hamburg, 2008). Der politische Erfolg bemisst sich darin, dass die Finanzierung einer gewünschten Maßnahme im Haushaltsplan untergebracht ist. Die Abrechnung wird im kameralen System nach meinem Eindruck eher selten näher betrachtet. Auf Landesebene sind die Jahresberichte der Rechnungshöfe voll von Fällen, in denen die Verwaltung Investitionen angenommen hat, ohne dass dies einer näheren Überprüfung standhielt. Das dürfte in den Kommunen mit kameralem Rechnungswesen nicht grundlegend anders sein. In der Doppik hat sich der Fokus auf die Rechnungslegung verlagert, weil die Vorschriften der

1 Obergruppen 93 bis 96 und 98 des bayerischen Gruppierungsplans, Anlagen 2 und 4, Bayerisches Staatsministerium des Innern, für Bau und Verkehr (2019a), sowie Kontenklasse 78 des Kommunalen Kontenrahmens Bayern, Anlagen 2 und 5, Bayerisches Staatsministerium des Innern, für Bau und Verkehr (2019b).

2 Zu den Ausgaben für Investitionen gehören auf der staatlichen Ebene Ausgaben für Baumaßnahmen (soweit sie nicht militärische Anlagen betreffen), für den Erwerb von beweglichen Sachen (soweit sie nicht als sächliche Verwaltungsausgaben veranschlagt werden oder soweit es sich nicht um Ausgaben für militärische Beschaffungen handelt), für den Erwerb von unbeweglichen Sachen, den Erwerb von Beteiligungen und sonstigem Kapitalvermögen, von Forderungen und Anteilsrechten an Unternehmen, von Wertpapieren sowie für die Heraufsetzung des Kapitals von Unternehmen, für Darlehen, für die Inanspruchnahme aus Gewährleistungen sowie für Zuweisungen und Zuschüsse zur Finanzierung von Ausgaben für die vorgenannten Zwecke, §10 Absatz 3 Nr. 2 Satz 2 Haushaltsgrundsätzegesetz (2017). Nach Nr. 5.7.2.3.4 der Standards Doppik des Gremiums zur Standardisierung des staatlichen Rechnungswesens (2020) sind bei hinreichender Wahrscheinlichkeit einer Inanspruchnahme aus Gewährleistungen dagegen Rückstellungen zu bilden, die Aufwand darstellen. Militärische Beschaffungen werden wie andere Vermögensgegenstände nach ihren Anschaffungs- oder Herstellungskosten abgegrenzt und sind deshalb überwiegend den Investitionen zuzurechnen. Für die Kameralistik gilt eine Wertgrenze von 5000 Euro, für die Doppik von 800 Euro.

kaufmännischen Rechnungslegung hierfür gemacht sind. Das Haushaltsrecht mit Voranschlag und Ermächtigungen sowie den mit den erforderlichen Schätzungen notwendigerweise verbundenen Ungenauigkeiten steht hierzu in einem latenten Spannungsverhältnis. Deshalb ist die Umstellung von der Kameralistik auf die Doppik Anlass, alle bisherigen vermeintlichen Gewissheiten einer Kontrolle zu unterziehen. Ungenauigkeiten in der Planung müssen „begradigt" werden. Der Rückgang der Investitionen könnte also – wie die Autor:innen dann ja auch einräumen – schlicht an einer höheren Genauigkeit in der Abgrenzung von Unterhaltungsaufwand und Investition liegen.

2. Im Rahmen einer jeden Haushaltsplanung ist entscheidend, in welchem Umfang Aufwendungen verursacht und Auszahlungen – auch für Investitionen – geleistet werden dürfen. Maßgeblich dafür ist zum ersten, ob der Ergebnisplan in Erträgen und Aufwendungen mindestens ausgeglichen sein muss. Hamburg hat sich z. B. zum Ziel gesetzt, seinen Gesamtergebnisplan ab dem Haushaltsjahr 2024 – bereinigt um konjunkturelle Effekte und Auswirkungen von außerordentlichen Notsituationen wie der COVID-19-Pandemie – so aufzustellen, dass die Erträge die Aufwendungen mindestens decken. Bis dahin ist übergangsweise noch ein jährlich sinkendes Defizit für den Gesamtergebnisplan zulässig. Für die Kommunen wurde der Haushaltsausgleich in den Ländern unterschiedlich geregelt. So wird teilweise zwischen ordentlichem und außerordentlichem Ergebnis unterschieden, um dann die Anforderungen an den Ausgleich auf das ordentliche Ergebnis,[3] beide jeweils für sich[4] oder beide zusammen zu beziehen.[5] Auch wird teilweise zugelassen, dass der Haushaltsplan zeitlich befristet nicht in Erträgen und Aufwendungen ausgeglichen wird, um dann unterschiedliche Anforderungen an den Ausgleich der dadurch entstehenden Fehlbeträge zu stellen.[6] Müssen nicht alle Aufwendungen eines Ergebnisplans durch Erträge ausgeglichen sein, fehlen auch Einzahlungen, die für Auszahlungen für Investitionen genutzt werden können.

Für die Betrachtung ist nämlich zweitens von Bedeutung, wie die Erträge zusammengesetzt sind, insbesondere welcher Anteil zahlungswirksam ist. Hier ist für Hamburg festzustellen, dass die Erträge weit überwiegend aus zahlungswirksamen Steuern, Gebühren und Bußgeldern bestehen. Auf der Aufwandsseite stehen dem in erheblichem Umfang nichtzahlungswirksame Abschreibungen und Zuführungen zu Rückstellungen gegenüber. Dies hatte seit Einführung der Doppik zur Folge, dass die Freie und Hansestadt Hamburg kein Liquiditätsproblem hatte. Die Steuerungsimpulse für politische Entscheidungen rühren aus den Grenzen her, die der Gesamtergebnisplan setzt, also aus den Erträgen. Die jährlich durch Abschreibungen nicht gebundenen Einzahlungen wurden vollständig zur Deckung von Auszahlungen für Investitionen veranschlagt.[7]

Für eine extensive Haushaltswirtschaft mit darüber hinausgehenden Investitionen war dagegen in Hamburg kein Raum mehr. Denn war es früher zulässig, Investitionen durch die Aufnahme von Krediten zu finanzieren, verbietet dies die sogenannte Schuldenbremse mittlerweile. Auch in den Kommunen wurden in den letzten Jahren verstärkt auf die Einhaltung der Regelungen zur Kreditaufnahme geachtet. Investitionen, die über den Ersatz für abgeschriebene Vermögensgegenstände hinausgehen, müssen deshalb aus den laufenden Einnahmen (Kameralistik) oder Einzahlungen (Doppik) finanziert werden können. Für die Steigerung der Investitionstätigkeit müssten deshalb Überschüsse im Ergebnisplan erzielt werden, sodass zahlungswirksamen Erträgen keine Aufwendungen und in der Folge auch keine konsumtiven Auszahlungen gegenüberstehen. Der mit der Schuldenbremse und einem Kreditaufnahmeverbot verfolgte politische Wille, mit den (Steuer-)Einnahmen von heute das Vermögen von morgen vorfinanzieren zu müssen, hat aber nichts mit der Doppik als Rechnungsstil zu tun. Der Ergebnisausgleich, also die Anforderung, auch den Vermögensverzehr zu verdienen, ist für alle Gebietskörperschaften eine erheblich größere Herausforderung als der Ausgleich von kameralen Einnahmen und Ausgaben ohne Einnahmen aus Krediten. Dies kann nur mit langen Umstellungsfristen gelingen. Viele Kommunen scheitern hieran schon unabhängig von außergewöhnlichen Notsituationen (siehe z. B. Sächsischer Rechnungshof, 2014). Vor diesem Hintergrund wäre für die in den Studien untersuchten Kommunen zu prüfen gewesen, ob die Ergebnispläne bereits ausgeglichen waren, wie sich die Erträge zusammengesetzt haben,

3 So z. B. §80 Absatz 2 Satz 2 Gemeindeordnung für Baden-Württemberg (2000).

4 Siehe z. B. §110 Absatz 4 Sätze 1 und 2 Niedersächsisches Kommunalverfassungsgesetz (2020).

5 So §63 Absatz 4 Kommunalverfassung des Landes Brandenburg (2019).

6 Siehe z. B. §24 Verordnung über das Haushalts-, Kassen- und Rechnungswesen der Gemeinden, der Landkreise und der Bezirke nach den Grundsätzen der doppelten kommunalen Buchführung (Kommunalhaushaltsverordnung-Doppik – KommHV-Doppik, 2019); §110 Absatz 5 Niedersächsiches Kommunalverfassungsgesetz (2020).

7 Siehe z. B. Haushaltsplan 2019/2020: Den im Gesamtergebnisplan veranschlagten „Aufwendungen aus Abschreibungen" in Höhe von jeweils etwa 625 Mio. Euro zuzüglich der „Abschreibungen auf Finanzanlagen und Wertpapiere des Umlaufvermögens" in Höhe von etwa 46 Mio. Euro in 2019 und 60 Mio. Euro in 2020 (= 671 bzw. 685 Mio. Euro) steht im doppischen Gesamtfinanzplan ein Saldo aus den Einzahlungen und Auszahlungen aus Investitionen in Höhe von 665 bzw. 689 Mio. Euro gegenüber. Hinzu kommen sogar noch jeweils über 220 Mio. Euro des gesondert ausgewiesenen Saldos der Einzahlungen und Auszahlungen aus gegebenen Darlehen. Der Gesamtergebnisplan und der doppische Gesamtfinanzplan sind Teil des Bandes „Gesamthaushalt 2019/2020" der Finanzbehörde Hamburg (2020, 31 ff.).

insbesondere ob sie weit überwiegend zahlungswirksam waren, und wie sich die Rahmenbedingungen für die Kreditaufnahme verändert haben, bevor man eine vermeintlich geringere Investitionstätigkeit dem Rechnungswesen anlastet und daraus am Ende auch noch ein Votum zur Einführung von EPSAS (European Public Sector Accounting Standards) ableitet.

## Erwiderung: Licht und Schatten der Doppik-Umstellung

In der Septemberausgabe 2020 des Wirtschaftsdiensts haben wir die Ergebnisse unserer drei empirischen Studien dargestellt (Christofzik et al., 2020).[1] Sie dokumentieren tendenziell einen Rückgang der Investitionen in deutschen Kommunen nach ihrer Umstellung auf die staatliche doppelte Buchführung (Doppik). In einer Replik kommentiert Stephan Stübner unsere Ergebnisse. Insbesondere bezweifelt er die Validität der verwendeten Daten, bemängelt die Ursachensuche und stellt unseren Ergebnissen die aus seiner Sicht positiven Erfahrungen mit der Doppik in Hamburg gegenüber. Wir nutzen diese Erwiderung für Erläuterungen.

*1. Validität der Daten*: Wenn wir von „Ausgaben“ sprechen, meinen wir Ausgaben. Stephan Stübner kritisiert, dass wir in unseren Studien kamerale Ausgaben mit doppischen Aufwendungen vergleichen würden. Eine solche Vermischung wäre in der Tat abwegig und wurde deshalb auch in keiner der drei Untersuchungen vorgenommen. Stattdessen verwenden wir durchgehend kamerale Ausgabendaten, auch für doppisch buchende Kommunen.[2] Dies ist aufgrund der finanzstatistischen Berichtspflichten möglich.[3] Für die Gemeinden und Gemeindeverbände

© Der/die Autor:in(nen) 2021. Open Access: Dieser Artikel wird unter der Creative Commons Namensnennung 4.0 International Lizenz veröffentlicht (creativecommons.org/licenses/by/4.0/deed.de).

Open Access wird durch die ZBW – Leibniz-Informationszentrum Wirtschaft gefördert.

1 Die drei empirischen Studien wurden unabhängig voneinander mithilfe quasi-experimenteller Methoden erstellt (Christofzik, 2019; Dorn et al., 2020; Raffer, 2020). Alle drei Studien analysieren, wie sich verschiedene Haushaltskennziffern nach der Doppik-Einführung in deutschen Kommunen verändert haben.

2 Der Begriff „laufender Sachaufwand“ hat hierbei nichts mit doppischen Aufwendungen zu tun, sondern umfasst als feststehender Begriff in der amtlichen Statistik neben den sächlichen Verwaltungsausgaben die Erstattungen an andere Bereiche. Den Begriff „Personalaufwand“ verwenden wir – entgegen den Aussagen in der Replik – nicht.

3 Vom Bund und den Ländern müssen gemäß §49b Haushaltsgrundsätzegesetz weiterhin Daten nach dem Gruppierungs- und Funktionenplan mindestens bis auf die Ebene der Dreisteller bereitgestellt werden (siehe auch die Regelungen im FPStatG).

**Prof. Dr. Désirée I. Christofzik** ist Professorin für öffentliche Finanzwirtschaft an der Hochschule des Bundes für öffentliche Verwaltung in Brühl.

**Florian Dorn** ist Vorstandsreferent am ifo Institut in München und Doktorand an der LMU München mit Forschungsschwerpunkten in der öffentlichen Finanzwirtschaft und politischen Ökonomie.

**Dr. Stefanie Gäbler** war wissenschaftliche Mitarbeiterin am Zentrum für öffentliche Finanzen und politische Ökonomie am ifo Institut in München.

**Christian Raffer** ist wissenschaftlicher Mitarbeiter am Forschungsbereich Infrastruktur, Wirtschaft und Finanzen des Deutschen Institut für Urbanistik (difu) in Berlin sowie Doktorand an der Freien Universität Berlin.

**Dr. Felix Rösel** ist wissenschaftlicher Mitarbeiter an der Dresdner Niederlassung des ifo Instituts mit Forschungsschwerpunkten in lokalen öffentlichen Finanzen und politischer Ökonomie.

bereiten die Statistischen Landesämter die Daten nach landesspezifischer und bundeseinheitlicher Gliederung auf. Dafür werden für Doppik-Kommunen Überleitungen vorgenommen, sodass die Haushaltsdaten ins kamerale Schema passen. Da eine eindeutige Zuordnung nicht in allen Fällen möglich ist, standen wir während des Forschungsprozesses in engem Austausch mit den Statistischen Ämtern der Länder oder des Bundes und haben teils durch Sonderauswertungen bereinigte Haushaltsdaten erhalten, die zwischen doppisch und kameral buchenden Kommunen vergleichbar sind.[4] Des Weiteren haben wir hierbei die vor der Doppik bereits bestehenden Unterschiede zwischen den umstellenden und nicht umstellenden Kommunen herausgerechnet (Differenzen-in-Differenzen-Ansatz; siehe Kugler et al., 2014).

*2. Mögliche Ursachen des Investitionsrückgangs*: In einer aktuellen Umfrage bestätigt die Mehrzahl der befragten Kommunen in Sachsen, dass die Doppik Auswirkungen auf ihre Investitionstätigkeit hat (Geißler et al., 2020). Etwa 85 % der Gemeinden heben in diesem Zusammenhang den Nexus von Investitionen und Abschreibungen hervor – auch Stephan Stüber betont dies in seinen Ausführungen. Viel spricht also für die Vermutung, dass die „Einpreisung“ von Abschreibungen eine Bremswirkung bei kommunalen Investitionen hat und die von uns gefundenen Effekte keine bloßen Artefakte der Buchungsdefinitorik sind.

Stephan Stüber führt weitere Gründe für eine geringere kommunale Investitionstätigkeit an, die ihre Berechtigung haben mögen, aber durch unser Studiendesign für die Ergebnisse keine Rolle spielen können. Wir nutzen aus, dass die Doppik zu unterschiedlichen Zeitpunkten in den einzelnen Kommunen eingeführt wurde und vergleichen die Entwicklung der kommunalen Finanzen vor und nach der Doppik-Einführung mit einer Kontrollgruppe von noch kameral buchenden Kommunen. Stephan Stübner bringt an, dass Kommunen aufgrund der Schuldenbremse ebenso verstärkt auf die Einhaltung von Kreditgrenzen achten und dies zu einem Rückgang von kreditfinanzierten Investitionen führen könne. Für die von uns betrachteten Kommunen in den Flächenländern könnte dies zunächst nur unter der Prämisse gelten, dass die Länder im Vorgriff auf die ab dem Jahr 2020 für sie geltende Schuldenbremse tatsächlich stärker auf die Kreditaufnahme ihrer Kommunen geachtet haben. Die Kommunen selbst werden von der Schuldenbremse nicht erfasst und können grundsätzlich weiterhin Kredite für Investitionen aufnehmen. Jedoch wären selbst dann gleichermaßen alle Kommunen betroffen – die doppisch buchenden Kommunen ebenso wie die Vergleichsgruppe. Daher kann die Schuldenbremse unsere Ergebnisse nicht erklären.[5]

Außerdem weist Stephan Stüber in seiner Replik darauf hin, dass veränderte Regelungen zum Haushaltsausgleich den Spielraum für Investitionen einschränken können. Er liefert stichhaltige Erklärungen, warum es eine deutlich größere Herausforderung sein kann, im doppischen Haushalt einen Ergebnishaushalt auszugleichen, als den Ausgleich kameraler Einnahmen und Ausgaben zu erreichen und räumt dabei ein, dass sich dies in geringeren Investitionen niederschlagen kann. Die veränderten Regelungen zum Haushaltsausgleich folgen aber zwangsläufig aus der Umstellung des Haushalts- und Rechnungswesens. Daher ist dies gerade eine Erklärung und keine Entkräftung der von uns beobachteten Effekte. Obwohl Stephan Stüber zu Beginn seiner Replik bezwei-

4 Während wir für unsere Studien und die dort betrachteten Aggregate auf vergleichbare Daten zurückgreifen konnten, zeigt sich für andere Zeitreihen ein deutlicher „Doppik-Bruch“. Im Sinne einer evidenzbasierten Wirtschaftspolitik wäre es daher wünschenswert, disaggregierte und vergleichbare Daten möglichst umfassend zur Verfügung zu stellen.

5 Auch empirisch lassen sich keine Auswirkungen auf den Schuldenstand feststellen (Christofzik, 2019).

felt, dass der Unterschied mit einem veränderten Entscheidungsverhalten zu tun hat, schreibt er schließlich, es könne „sicher auch festgehalten werden, dass die Doppik das Entscheidungsverhalten verändert hat."

*3. Andere Erfahrungen*: Unsere Ergebnisse zeigen, dass die Doppik wie gewünscht das kommunale „Tafelsilber" besser schützt. Auch haben wir in unserem Artikel weitere Vorteile der Doppik herausgestellt. Der Preis hierfür sind jedoch höhere Verwaltungsausgaben. Wie bei jeder politischen Maßnahme stellt sich nüchtern die Frage, ob die Vorteile der Doppik ihre Kosten aufwiegen. Dies dürfte regional sehr unterschiedlich sein. Eine unter dem Strich positive Bilanz der Doppik, die Stephan Stüber in seiner Replik für die Großstadt Hamburg mit personalstarker Finanzbehörde und zahlreichen öffentlichen Beteiligungen ziehen mag, kann für kleine Gemeinden im ländlichen Raum ganz anders ausfallen. Stephan Stübner stellt selbst für Hamburg fest, dass das „neue Haushaltswesen (…) Politik und Verwaltung einiges abverlangt" hat. Zahlreiche Kommunen berichten von höheren Personal- und Verwaltungsausgaben sowie hohen Implementierungskosten durch die Anforderungen der kommunalen Doppik (vgl. Böhme et al. 2013, Geißler et al. 2020). Die Doppik ist in dieser Hinsicht teurer als die Kameralistik. In 80 % der befragten sächsischen Kommunen hat die Doppik den Fachkräftemangel im öffentlichen Dienst weiter verschärft (Geißler et al., 2020). Es verwundert deshalb nicht, dass in den beiden Bundesländern mit Wahlfreiheit – Bayern und Thüringen – die Doppik alles andere als ein Kassenschlager ist. Gerade einmal 5 % aller kreisangehörigen Gemeinden in diesen Ländern haben sich im Lauf der Zeit für die Doppik entschieden, 95 % buchen weiter kameral. In Bayern sind die fränkischen Kommunen Königsfeld, Niederwerrn und Selb von der Doppik sogar wieder zur Kameralistik zurückgekehrt oder bereiten dies vor.[6]

6 Vgl. „Zurück in die Zukunft": https://www.frankenpost.de/inhalt.selb-zurueck-in-die-zukunft.759beb5b-1cdb-48e5-a01b-36207806008e.html; „Rückumstellung des Rechnungswesens": http://www.hochfranken-live.de/index.php/aus-dem-rathaus/6300-aus-dem-stadtrat-notiert-31.html.

### Fazit

Die Erfahrungen mit der Doppik sind so bunt und unterschiedlich wie die kommunale Familie selbst. Umfragen zeigen eindrücklich Licht und Schatten. Die Erfahrung einer Kommune kann daher nicht verallgemeinert werden. Unsere drei Studien betrachten deshalb systematisch alle Kommunen eines Bundeslandes (Dorn et al., 2020; Raffer, 2020) oder die aggregierten Gemeindedaten für alle deutschen Flächenländer (Christofzik, 2019). Dabei haben wir die Vergleichbarkeit der Daten bestmöglich sichergestellt. Durch das Studiendesign können wir die beobachteten Unterschiede in den Haushaltskennziffern der Umstellung auf die doppelte Buchführung zuschreiben. Zahlreiche Argumente sprechen dafür, dass die Doppik zu niedrigeren Investitionen führen kann. Umfassende Reformen wie die Umstellung des Rechnungswesens verlangen nach wissenschaftlich fundierten Analysen, um evidenzbasiert Entscheidungen treffen zu können.
